# Pulsed-laser irradiation of multifunctional gold nanoshells to overcome trastuzumab resistance in HER2-overexpressing breast cancer

**DOI:** 10.1186/s13046-019-1305-x

**Published:** 2019-07-12

**Authors:** Toni Nunes, Thomas Pons, Xue Hou, Khanh Van Do, Benoît Caron, Marthe Rigal, Mélanie Di Benedetto, Bruno Palpant, Christophe Leboeuf, Anne Janin, Guilhem Bousquet

**Affiliations:** 1 Université-Paris-Diderot, Sorbonne-Paris-Cité, Laboratoire Pathologie, UMR-S942, F-75010 Paris, France; 2 INSERM, U942 Paris, France; 3 LPEM, ESPCI Paris, PSL Research University, CNRS, Sorbonne Universités, UPMC, F-75005 Paris, France; 4 Laboratoire de Photonique Quantique et Moléculaire, CentraleSupélec, Ecole Normale Supérieure Paris-Saclay, Université Paris Saclay, CNRS UMR 8537, 3 rue Joliot Curie, F-91190 Gif-sur-Yvette, France; 5 ALIPP6, Institut des Sciences de la Terre de Paris UMR 7193, CNRS, Sorbonne Université, F-75005 Paris, France; 6 AP-HP-Hôpital Avicenne, Service-Pharmacie-Paris, Paris, France; 7 Université Paris 13, F-93430 Villetaneuse, France; 8 AP-HP-Hôpital Saint-Louis, Laboratoire-Pathologie-Paris, Paris, France; 9 AP-HP-Hôpital Avicenne, Service-Oncologie–Paris, Paris, France

**Keywords:** Functionalized gold nanoparticles, HER2-overexpressing breast cancer, Trastuzumab resistance, Resistance reversion, Photothermal therapy, Femtosecond laser

## Abstract

**Background:**

HER2-overexpressing metastatic breast cancers are challenging practice in oncology when they become resistant to anti-HER2 therapies such as trastuzumab. In these clinical situations, HER2-overexpression persists in metastatic localizations, and can thus be used for active targeting using innovative therapeutic approaches. Functionalized gold nanoparticles with anti-HER2 antibody can be stimulated by near-infrared light to induce hyperthermia.

**Methods:**

Here, hybrid anti-HER2 gold nanoshells were engineered for photothermal therapy to overcome trastuzumab resistance in HER2-overexpressing breast cancer xenografts.

**Results:**

When gold nanoshells were administered in HER2-tumor xenografts, no toxicity was observed. A detailed pharmacokinetic study showed a time-dependent accumulation of gold nanoshells within the tumors, significantly greater with functionalized gold nanoshells at 72 h. This enabled us to optimize the treatment protocol and irradiate the mice when the anti-HER2 gold nanoshells had accumulated most in the tumors. After weekly injections of anti-HER2 gold nanoshells, and repeated irradiations with a femtosecond-pulsed laser over four weeks, tumor growth was significantly inhibited. Detailed tissue microscopic analyses showed that the tumor growth inhibition was due to an anti-angiogenic effect, coherent with a preferential distribution of the nanoshells in tumor microvessels. We also showed a direct tumor cell effect with apoptosis and inhibition of proliferation, coherent with an immune-mediated targeting of tumor cells by anti-HER2 nanoshells.

**Conclusion:**

This preclinical study thus supports the use of anti-HER2 gold nanoshells and photothermal therapy to overcome trastuzumab resistance in HER2-overexpressing breast cancer.

**Electronic supplementary material:**

The online version of this article (10.1186/s13046-019-1305-x) contains supplementary material, which is available to authorized users.

## Background

Breast cancer is the most common cancer in women, and the leading cause of mortality among young women [1]. Fifteen to 20 % of breast cancers overexpress the human epidermal growth factor receptor-2 (HER2) [2], predicting response to anti-HER2 treatments [3]. Trastuzumab, the leading anti-HER2 drug [4], is a humanized monoclonal antibody that binds with high affinity and specificity to the extracellular domain of HER2. Trastuzumab-based chemotherapies have dramatically improved the prognosis of women with HER2-overexpressing metastatic breast cancers, with a median overall survival of 56 months and 50% long-term survivors with possible chemo-curability [5]. However, 50% of women develop resistance to trastuzumab [5]. The mechanisms of resistance to anti-HER2 therapies involve either mutations of the downstream signaling pathways, or alternative survival pathways that bypass the HER2 blockade [6]. In these clinical situations, HER2-overexpression persists in metastatic localizations in 70 to 80% of cases [7].

Over the past two decades, nanotechnologies have aroused great interest for biomedical applications, including drug delivery, with limited toxicity to normal tissues [8]. For cancer treatment with cytotoxic drugs, applications of this type still exist, for instance liposomal doxorubicin to avoid cardiotoxicity [9] or albumin-linked paclitaxel to decrease neurotoxic effects [10]. Gold nanoparticles are innovative tools for cancer treatment [11], particularly because they can be stimulated by near-infrared light to induce physical hyperthermia [12, 13].

**Fig. 1:**
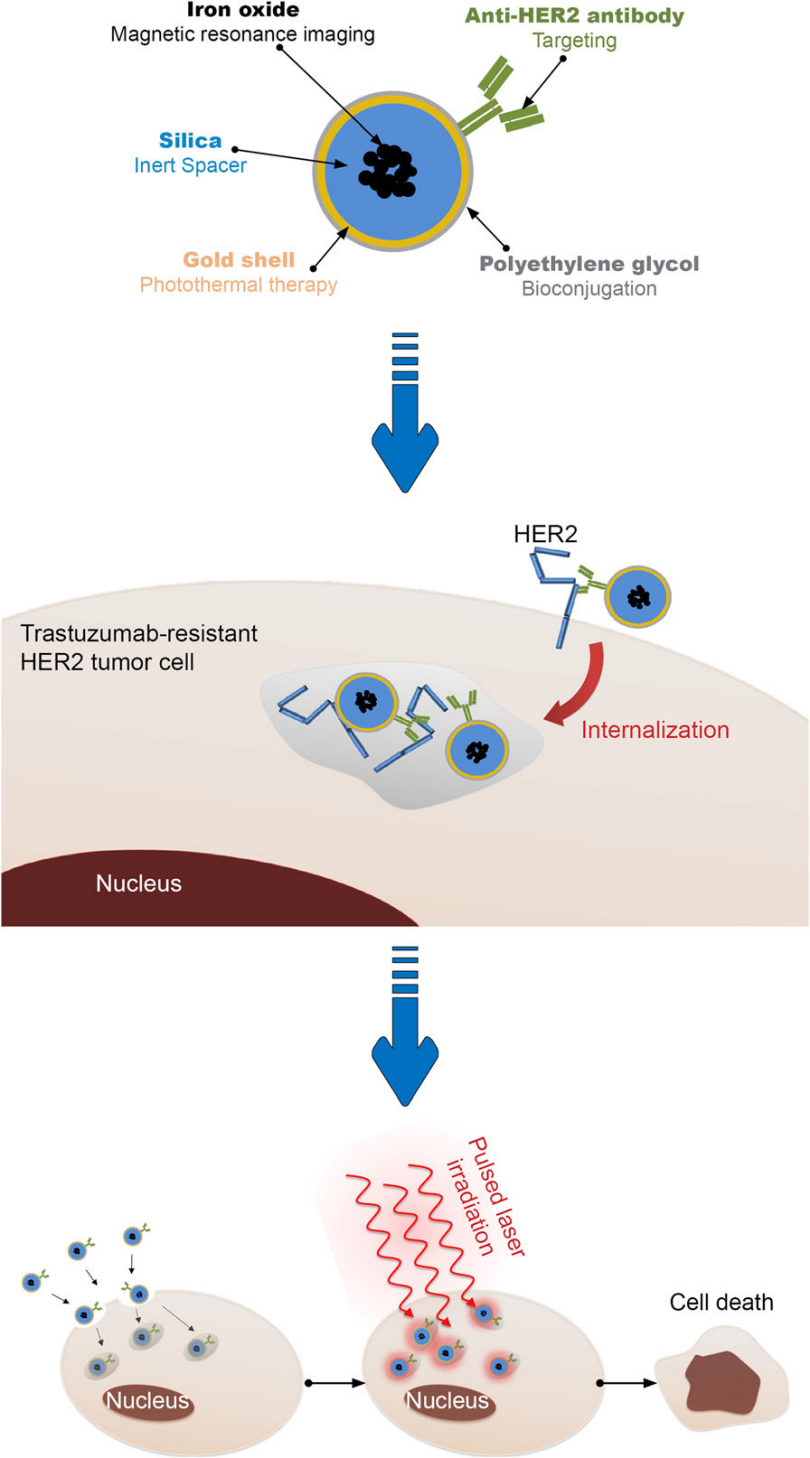
Illustration of the proposed multifunctional anti-HER2 gold nanoshell with HER2 receptor targeting and photothermal therapy

In this study we engineered a hybrid gold nanoshell, functionalized with an anti-HER2 antibody, to overcome trastuzumab resistance in HER2-overexpressing breast cancer (Fig.1).

## Methods

### Synthesis of iron oxide/silica/gold core/shell nanohybrids

The materials used were oleic acid (OA), oleylamine (OAm), 1,2-dodecanediol, tetraethoxysilane (TEOS), mercaptopropyltrimethoxysilane (MPTMS), sodium dodecylsulfonate (SDS), ammonia solution (28 wt% in water), Tetrakis (hydroxymethyl) phosphonium chloride (THPC) solution (80 wt% in water), and polyvinylpyrrolidone K12 (PVP), purchased from Sigma-Aldrich. HS-PEG5000-COOH was purchased from Iris Biotech. Anti-human HER2 rabbit polyclonal antibody (RB-9040-P) was purchased from Fisher Scientific.

First, iron oxide nanoparticles were synthesized from iron acetylacetonate precursors [14]. Briefly, 2 mmol Fe (acac)_3_ were mixed with 2 g of 1,2-dodecanediol, 6 mmol of oleic acid, 6 mmol of oleylamine and 20 mL of benzyl ether in a three-neck flask. The solution was degassed at 40 °C under vacuum for 15 min. It was then heated under argon at 200 °C for 1 h, then at 260 °C for 30 min. The nanoparticles were precipitated with ethanol and re-suspended in 10 mL of hexane. In a second regrowth step, 8 mL of this solution were mixed with 4 mmol of Fe (acac)_3_, 4 g of 1,2-dodecanediol, 4 mmol of oleic acid, 4 mmol of oleylamine and 40 mL of benzyl ether in a three-neck flask. The solution was degassed at 40 °C under vacuum for 15 min. It was then heated under argon at 200 °C for 1 h, then at 260 °C for 30 min. The resulting nanoparticles were precipitated with ethanol and re-dispersed in 10 mL of hexane.

For the encapsulation of iron oxide in silica, 660 μL of the iron oxide nanoparticle solution were precipitated in ethanol and re-suspended in 200 μL of chloroform, 80 μL of TEOS and 20 μL of MPTMS. 2 mL of a 8.6 mM SDS aqueous solution were added and the mixture was sonicated for 15 min (Vibracell sonicator). 12 mL of water and 100 μL of ammonia (28 wt% in water) were added and the solution was stirred overnight. The Fe_3_O_4_/SiO_2_ nanoparticles were isolated by 2 rounds of centrifugation (20 min, 18,000 g) and re-suspended in 1 mL of ethanol. Growth of the gold shell followed a protocol derived from those used by Pham et al. [15] and Ji et al. [16]. Gold seeds were synthesized following previously described protocols [17]. Briefly, 91 mL of water were mixed with 3 mL of NaOH 0.2 M and 24 μL of THPC (80 wt% in water) and stirred for 5 min. 6 mL of HAuCl4 solution (20 mM) were then added dropwise and the solution was stirred at room temperature for 15 min. The gold seed solution was then stored at 4 °C and matured for > 2 weeks.

Poly (1-vinylimidazole-co-vinyltrimethoxysilane) (PVIS) was synthesized as previously described [16]. Briefly, 50 μL of Fe_3_O_4_/SiO_2_ and 5 mg of PVIS were diluted in 7 mL of methanol, then maintained at 60 °C for 2 h. The nanoshells were washed by 3 rounds of centrifugation (10 min, 18,000 g) and re-suspended in 1 mL of water. 10 mL of the gold seed solution were added and the solution was stirred for 30 min. The excess gold seeds were eliminated with 2 rounds of centrifugation (5 min, 18,000 g) and the particles were re-suspended in 1 mL of water.

A potassium carbonate/gold chloride growth solution was prepared according to a protocol described by Pham et al. [15]. 250 μL of the resulting gold seeded-Fe_3_O_4_/SiO_2_ solution were mixed with 6 mL of gold growth solution, and 90 μL of PVP (0.35 wt% in water). 18 μL of formaldehyde solution (37 wt% in water) were then added and the solution was stirred for 6 h at room temperature. The Fe_3_O_4_/SiO_2_/Au particles were purified by 2 rounds of centrifugation.

Then, Fe_3_O_4_/SiO_2_/Au particles obtained in the previous step were re-suspended in 1 mL of PBS (pH 7.4) with 1 mg of HS-PEG5000-COOH and stirred overnight. The particles were purified by 2 rounds of centrifugation and re-suspended in 1 mL of PBS. 2 mg of EDC.HCl and 2 mg of NHS were dissolved in DMSO, immediately added to 500 μL of the Fe_3_O_4_/SiO_2_/Au solution and stirred for 15 min at room temperature. The nanoshells were purified by 2 rounds of centrifugation, re-suspended in 500 μL of PBS, mixed with 33 μL of anti-HER2 polyclonal antibody (0.2 mg/mL) and stirred for 1 h. The nanoshells were purified by 2 rounds of centrifugation and re-suspended in 500 μL of PBS. To characterize the nanoshells obtained, absorbance spectroscopy was performed with a Shimadzu UV-Vis-NIR UV-3600 spectrophotometer. Transmission electron microscopy was performed with a JEOL 2010 microscope to determine particle concentration and characterize particle morphology and size. The hydrodynamic diameter and surface charge (zeta potential) of the synthesized particles were measured by dynamic light scattering (Malvern Zetasizer Nano-ZS). For this study, we term the functionalized nanoparticles “anti-HER2 gold nanoshells (GNs)”.

### Human cancer cell lines

Two breast cancer cell lines were used, BT474 with HER2 overexpression and MDA-MB-231 which does not overexpress HER2. The cell lines were obtained from ATCC. These cells were cultured at 37 °C under normoxic conditions (20% of O2 and 5% of CO2) in RPMI 1640 medium supplemented with 10% of fetal calf serum and 1% antibiotics. To obtain a trastuzumab-resistant BT474 cell line, we exposed it to increasing sub-toxic concentrations of trastuzumab over a period of six months. The cell line was maintained viable at a concentration of 10 μg/mL. After exposure to trastuzumab was discontinued, trastuzumab resistance persisted after 10 passages, since IC_50_ was not reached. We term this resistant cell line “BT474-R”.

### Assessment of trastuzumab resistance on the BT474-R cell line

To assess the resistance of the BT474-R cell line towards trastuzumab, BT474 cells were seeded in 96-well tissue culture plates at a density of 5 × 10^3^ cells per well. After 24 h of incubation, the cells were exposed to increasing doses of trastuzumab (0 to 100 μg/mL) for 72 additional hours. Cell viability was determined by the colorimetric conversion of yellow, water-soluble tetrazolium MTT (3-[4, 5-dimethylthiazol-2-yl]-2,5-diphenyl-tetrazolium-bromide; Sigma), to purple, water-insoluble formazan. After incubation for 4 h at 37 °C with 0.4 mg/ml of MTT, the cells were placed in 0.1 ml of DMSO, and the absorbance was measured at 560 nm using a Fluostar Optima microplate reader (BMG LabTech). Experiments were performed in triplicate, untreated cells being used as positive controls, and trastuzumab-containing medium without cells as a negative control.

### *HER2* gene copy number

Droplet digital PCR was performed to assess the copy number of the *HER2* gene in BT474-R and MDA231 cell lines. DNA was extracted from both cell lines using the QIAamp® DNA Mini-Kit (Qiagen). DNA quality was assessed by spectrometric assay (NanoDrop® ND-1000, Thermo scientific). Each droplet digital PCR assay was performed according to the MIQE guidelines (minimum information for publication of quantitative real-time PCR experiment) and conducted in triplicate [18]. Reagent mixes (with Hs00223586_cn ERBB2 as the primer and TaqMan® Copy Number Reference Assay, human, RNase P, Life Technologies) were prepared using standard Taqman primer/probe chemistry with a 2 X ddPCR Mastermix (BioRad, Laboratories), a 20 X primer/probe (900/250 nM), and 5 μL of sample DNA template in a final volume of 20 μL. The reagent mixture was loaded into an eight-channel droplet generator (BioRad, Laboratories). 70 μL of droplet generation oil were loaded for each channel and after generation of water-in-oil droplets the droplets were transferred to a 96-well PCR plate and placed in a Biorad thermocycler. An initial denaturation step (95 °C, 10 min) was followed by 45 cycles at 95 °C for 15 s and at 60 °C for 1 min. The PCR products were streamed through a droplet reader and the results were analysed using QuantaSoft software (BioRad Laboratories). All droplets were gated on the basis of detector peak width to exclude doublets or triplets.

### *HER2* mRNA expression level

The *HER2* mRNA expression level was assessed using real-time quantitative RT-PCR. Total RNA was extracted from both cell lines using the RNeasy-Mini-Kit (Qiagen) and processed for reverse transcription. RNA quality was assessed by spectrometric assay (NanoDrop® ND-1000, Thermo scientific). The qPCR reactions were performed using fluorescent probes on a CFX96 Real Time System (Bio-Rad) and the gene expression level was assessed, using Hs01001580_m1 (ERBB2, Life Technologies) as the primer. The reference gene was human *TBP* with the primer Hs99999910_m1 (Life Technologies), a blank sample (no cDNA) was included, and experiments were performed in triplicate, with each sample in duplicate on the PCR plate. The results were expressed as 2^-ΔΔCq^ (relative quantification).

### HER2 immunohistochemistry assay

For each of the two cell lines, BT474-R and MDA231, a pellet was obtained after centrifugation of cultured cells. It was then formalin-fixed and paraffin-embedded. HER2 expression was assessed on 5 μm-thick paraffin sections with an indirect immunoperoxydase method using rabbit anti-Human HER2 (dilution 1:100, clone SP3, Spring Bioscience) as the primary monoclonal antibody. Systematic controls were the absence of primary antibody and the use of an irrelevant primary antibody of the same isotype. Tissue sections were analysed under an Olympus AX 70 microscope with a 0.344-mm^2^ field size at X400 magnification. Analyses were performed by two pathologists independently (GB, AJ).

### Assessment of trastuzumab binding to HER2 receptors

The ability of trastuzumab to efficiently bind to HER2 membrane receptors was assessed on the BT474-R and MDA-MB-231 cell lines. The two cell lines were grown separately on culture slides (BD Falcon™). Five micrograms of commercial trastuzumab (Roche) coupled with Alexa Fluor 488 fluorophore (using APEX™ Alexa Fluor™ 488 Antibody Labeling Kit, Invitrogen) were diluted in 300 μL of PBS and incubated for 1 h with each type of human cancer cell line. Then, the PBS was removed and the cells were rinsed to remove unbound antibodies. The cells were formalin-fixed, the nuclei were stained with DAPI (Abcam) and fluorescence staining was observed at 400x magnification. The experiment was conducted five times independently, and a minimum of 100 cells were analysed.

### Assessment of nanoshell binding to HER2 receptors

We assessed the ability of anti-HER2 nanoshells to efficiently bind to HER2 receptors on the surface of BT474-R cells. BT474-R and MDA231 cell lines were grown separately on culture slides (BD Falcon™) and incubated for 1 h with 10^10^ anti-HER2 nanoparticles. Then the suspension was removed and cells were rinsed to remove unbound nanoshells. The cells were formalin-fixed and stained with DAPI (Abcam). The binding of nanoshells to the cells was assessed by dark field light microscopy at 400x magnification using an Olympus AX 70 microscope. The experiment was conducted five times independently, and a minimum of 100 cells were analysed.

### Assessment of cell viability

For cell viability assessment, the BT474-R and MDA231 cell lines were seeded separately in 96-well tissue culture plates at a density of 5 × 10^3^ cells per well. After 24 h of incubation, the cells were exposed to increasing numbers of nanoshells (10^12^, 10^13^ or 10^14^ nanoshells) for 24 additional hours. Cell viability was determined by the colorimetric conversion of yellow, water-soluble tetrazolium MTT (3-[4, 5-dimethylthiazol-2-yl]-2,5-diphenyl-tetrazolium-bromide; Sigma), to purple, water-insoluble formazan. After incubation for 4 h at 37 °C with 0.4 mg/ml of MTT, the cells were placed in 0.1 ml of DMSO, and the absorbance was measured at 560 nm using a Fluostar Optima microplate reader (BMG LabTech). Experiments were performed in triplicate, untreated cells being used as positive controls, and a nanoshell-containing medium without cells as a negative control.

### Xenograft models with HER2 over-expressing cancer cell lines

Female, nu/nu athymic mice of NMRI background (R. Janvier) aged 6 weeks, were xenografted sub-cutaneously with the BT474-R cell line. The mice were housed in the animal facility of the University Institute of Haematology, Paris, France. The University Institute Ethics Committee Board for experimental animal studies approved this study (N°2012–15/728–0115).

For the initial xenografts, 10^6^ BT474 cells were injected subcutaneously into the back under isoflurane anaesthesia (*n* = 5 mice). For each passage, 10 mm^3^ of tumor fragments were xenografted into 5 mice. A daily clinical score was recorded and tumor growth was measured weekly until tumor weight reached the ethically recommended limit of less than 10% of mouse weight (2010/63/EU Directive of the European Parliament and Council of 22nd September 2010 on the protection of animals used for scientific purposes; Official Journal of European Union L 276/33). The tumor volume was calculated as V = L x l^2^/2, L being the larger diameter (length), l the smaller (width). For each mouse, the tumors and the different organs were systematically analysed. Tumors were dissected and divided into four parts: one part was immediately snap-frozen in liquid nitrogen, one part was formalin-fixed (fixing agent AFA, CARLO ERBA Reagents) and paraffin-embedded (Tissue -Tek® Paraffin Wax), one part was glutaraldehyde-fixed and Epon resin-embedded, and a fourth part was used for the next passage. Mice were used for our studies when the tumors reached 150 mm^3^.

### Pharmacokinetic and bio-distribution studies

Gold nanoshells were administered via a 200-μL tail vein injection of 3.5 × 10^13^ nanoshells suspended in 0.9% aqueous sodium chloride solution (Versylene® Fresenius). Two different types of gold nanoshells were administered: functionalized anti-HER2 and non-functionalized gold nanoshells. For each type, a group of mice (*n* = 5) was used for pharmacokinetic studies and another group for bio-distribution assessment on MRI. For pharmacokinetics, blood samples were taken at 10 min and then at 3, 24, 48 and 72 h after administration. A biopsy of the xenografted tumor was performed at 24 h. The total gold content in tissues was quantified using ICP-MS. For the MRI exploration, we carried out a kinetic study of bio-distribution, with image acquisitions from 0 to 1 h, then at 3, 6, 24 and 72 h after administration.

### Inductively coupled plasma-mass spectrometry (ICP-MS)

The total gold content in mouse tissues (tumor, liver, spleen and blood) was quantified using ICP-MS facilities on the ALIPP6 platform of ISTeP UMR 7193, Sorbonne University, Paris, France. After excision, tissue samples were stored at − 80 °C until preparation for ICP-MS measures. Organs were digested in closed vials with 3 mL of nitric acid (HNO_3_, Normatom® 67%) at 70 °C for 2 h on a heat block (DigiPREP Jr. SCP Science) and then dried in open vials at 80 °C for 8 h. The volume of each sample was adjusted according to the initial sample weight with a deionized water solution (MilliQ Millipore®) acidified with 2% nitric acid, and then analysed for gold content using the ICP-MS-MS system (Agilent Technologies 8800 triple quadrupole). A multi-element solution (Tune A) was used to optimize the initial parameters of the instrument to obtain the best sensitivity and a minimum drift of the signal. Gold element (Au) was measured at an atomic mass of 197, with MS-MS no-gas mode, and an integration time between 0.1 and 0.51 ms over an analytical period of more than one year. Data acquisition was performed in counts per second (cps) with three replicates for each sample. The instrument was calibrated with a standard mono-element 1000 ppm (ppm) of gold calibration solution (PlasmaCAL SCP science) using concentration values between 1 and 100 parts per billion (ppb). Tissue from untreated mice were analysed and used as controls.

### Magnetic resonance imaging (MRI)

MRI studies were carried out at 25 °C using a micro-MRI 7 Teslas (Bruker, Advance II, Resolution below 100 μm), dedicated to small animals. We used a Bruker acquisition coil with an internal 40-mm diameter, with a mouse-specific restraint system containing an anaesthetic mask. MRI imaging of GNs samples was conducted and the relaxation rates were calculated. Then, the mice were positioned in a suitable non-magnetic harness equipped with a facial mask, and were anaesthetized with isofluorane at a dose of 1.5% and an oxygen-air mixture (1:2, 0.9 L/min). The physiological parameters of temperature and respiration rate of the mice were monitored throughout the acquisition with a plastic sensor positioned on the thorax and a physiological monitor for the respiratory value (SA Instruments Inc.). Image acquisition was carried out at time 0. Then, after intravenous administration of 3.5 × 10^13^ nanoshells, a continuous acquisition of images from 0 to 1 h and then acquisitions at 3, 6, 24 and 72 h were performed. The images were recorded using the acquisition software Bruker Paravision 5.1, and then analysed.

### Analysis of gold nanoshells in tissues

The mice were euthanized at 48 or 72 h after administration. At the time of euthanasia, organs and tumors were systematically removed and analyzed as described. One fragment was formalin-fixed and paraffin-embedded, another fragment was snap-frozen at − 80 °C, and a third was fixed with 2% glutaraldehyde in 0.1 M sodium cacodylate buffer and Epon resin-embedded. Blood samples were stored at − 80 °C.

Tissue toxicity was assessed for each organ on H&E colored, 2 μm-thick paraffin sections. The histo-pathological features and location of the nanoshells were analyzsed by two pathologists independently (AJ, GB).

Dark field light microscopy enabled direct visualization of the gold nanoshells on uncolored 5 μm-thick paraffin sections at × 100 magnification. This also enabled an assessment of nanoshell distribution in endothelial or tumor cells by combining CD31 (rat anti-Mouse CD31 antibody, dilution 1:50, clone SZ31, Dianova), or Cytokeratin (rabbit anti-Human Cytokeratin antibody, dilution 1:50, clones AE1/AE3, Dako) immunostaining with dark field analysis.

### Transmission electron microscopy (TEM)

Organ samples were immediately cut into 1-mm^3^ pieces and fixed with 2% glutaraldehyde in 0.1 M of sodium cacodylate buffer. After ethanol-dehydration and Epon-embedding, semi-thin sections were prepared. Ultrathin sections (150 nm) were obtained on selected zones, stained and analyzed on a Hitachi H-7650 TEM microscope at 80 kV.

### Photothermal therapy

The BT474-R and MDA231 cell lines were seeded separately on 48-well tissue culture plates at a density of 5 × 10^4^ cells per well. After 48 h of incubation, the cells were exposed to 10^12^ anti-HER2 nanoshells for 2 h. After rinsing, each well was irradiated using a Ti/sapphire femtosecond laser (Hurricane, Spectra Physics Lasers), which provided 120 femtosecond FWHM pulses (wavelength 800 nm, maximum energy 1 mJ/pulse, repetition rate 1 kHz) for 5 min. Cell viability was determined by cell counts. Experiments were performed in triplicate. Untreated cells and irradiated cells without nanoshells were used as controls. To check the photothermal stability of the nanoshells under irradiation, absorbance spectroscopy was performed with an Agilent Cary 5000 spectrophotometer, before and after 30 min of continuous pulsed laser irradiation.

Anti-HER2 gold nanoshells were administered via 200-μL tail vein injection of 5 × 10^12^ nanoshells in the mice with two tumors (*n* = 10). After 72 h, only one tumor was irradiated using a Ti/sapphire femtosecond laser (wavelength 800 nm) for 30 min, and the temperature of the irradiated area was monitored during photothermal treatment using a thermal camera (FLIR Systems, Inc.). The apoptosis analysis of cancer cells is also needed for photothermal therapy. This treatment procedure was repeated once a week for 4 weeks. Treated mice with non-functionalized gold nanoshells were used as the controls. Tumor volume was assessed twice a week. At the time of euthanasia, organs and tumors were systematically removed and analysed.

To assess the photothermal effect of the Anti-HER2 gold nanoshells on irradiated tumors, necrosis areas were quantified on 2 μm-thick scanned H&E coloured sections using NDP.view2 Viewing software. Vessels were quantified on 5 μm-thick sections after CD31 immunostaining on 5 different non-necrotic fields at 200x magnification, and expressed as the number of positive cells/High Power Field (HPF). Proliferating and apoptotic cells were quantified on 5 μm-thick sections after Ki67 and Cleaved caspase-3 immunostainings on 5 different non-necrotic fields at 400x magnification, and expressed as percentages of positive cells/HPF. Staining was performed using an indirect immunoperoxydase method with a rat anti-Mouse CD31 antibody (dilution 1:50, clone SZ31, Dianova), a mouse anti-Human Ki67 antibody (dilution 1:50, clone MIB-1, Dako), and a rabbit anti-Human Cleaved caspase-3 antibody (dilution 1:50, clone 5A1E, Cell Signaling Technology) as primary antibodies. Systematic controls were the absence of primary antibody and the use of an irrelevant primary antibody of the same isotype. Quantifications were performed using cellSens Dimension software (Olympus). Non-irradiated tumors were used as controls. To assess endothelial cell apoptosis, we performed a double CD31 and Cleaved caspase-3 immunostaining using a rat anti-Mouse CD31 antibody (dilution 1:50, clone SZ31, Dianova), and a rabbit anti-Human Cleaved caspase-3 antibody (dilution 1:50, clone 5A1E, Cell Signaling Technology). Non-irradiated tumors were used as controls.

### Statistical analysis

Data were expressed as mean ± standard deviation (SD) or standard error (SE). Comparisons between the groups were made using Student’s t-test, and for more than two groups, ANOVA was used. A *P* value < 0.05 was considered to indicate statistical significance.

## Results

### Preparation and characterization of gold nanohybrids

**Fig. 2:**
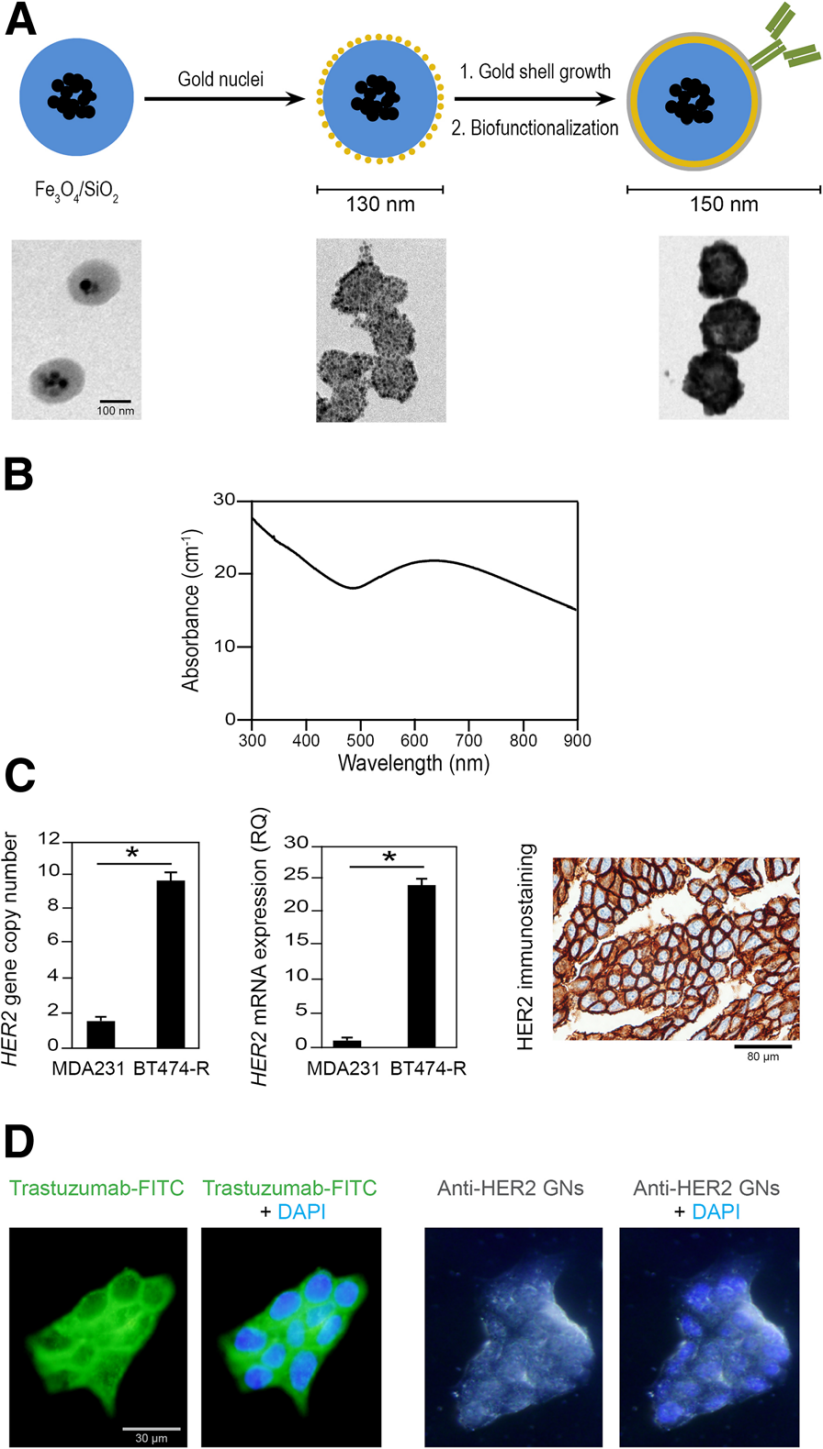
**a** Diagram showing steps in anti-HER2 gold nanoshell (GN) synthesis with corresponding TEM images. **b** UV-Vis absorption spectra of anti-HER2 GNs. **c** *HER2* gene copy number, mRNA expression and immunostaining (brown) for BT474-R cell line (* *P* < 0.05). **d** Immunostaining of BT474-R cells incubated with trastuzumab-FITC (green, left panel). Dark field images of BT474-R cells incubated with anti-HER2 gold nanoshells (right panel)

Hydrophobic iron oxide nanocrystals were synthesized using solvothermal strategies. They were grouped into size-controlled clusters using an emulsion-evaporation scheme together with anionic surfactants and silica precursors. These precursors were hydrolyzed and condensed using ammonia. The Fe_3_O_4_/SiO_2_ nanoparticles were then purified by centrifugation. TEM observations showed that each nanoparticle contained between 6 and 10 iron oxide nanocrystals. The nanoparticles were then functionalized with a poly-(vinylimidazole-co-silane) polymer (PVIS) to bind small gold nuclei to the silica surface. Gold could then be grown from these surface-bound gold clusters to yield a continuous gold shell around the initial Fe_3_O_4_-silica-PVIS particles (Fig.2a). After synthesis, the nanoshells were coated with bifunctional polyethylene glycol (PEG) polymers, bearing a dithiol group at one end for stable anchoring onto the gold surface, and a carboxylic acid group at the other end for further bio-conjugation. After purification, the nanoshells were conjugated to rabbit anti-human HER2 polyclonal antibody using N-Hydroxysuccinimide ester activation of the surface carboxylate groups. We term these functionalized nanoparticles “anti-HER2 gold nanoshells (GNs)”.

After synthesis, the characteristics of the nanoparticles were analyzed using dynamic light scattering, spectrophotometry and electron microscopy. The size of the non-functionalized gold nanoshells was 128 ± 13 nm with a zeta potential of − 32.1 ± 0.5 mV. For the anti-HER2 gold nanoshells, the size was 148 ± 12 nm and − 7.3 ± 0.5 mV, respectively. TEM images showed that the particles were monodispersed in a physiological medium (aqueous sodium chloride solution) with colloidal stability. Using a 7-Teslas MRI, the relaxivity at 25 °C was 48.5 mM-1 s-1 for the non-functionalized gold nanoshells and 44.0 mM-1 s-1 for the anti-HER2 gold nanoshells (Additional file 1: Figure S1A and B). Both non-functionalized and anti-HER2 gold nanoshells absorb in a broad visible-near-infrared window (Fig. 2b), with no difference before irradiation or after 30 min of continuous pulsed laser irradiation (Additional file 1: Figure S1C).

### Development of an HER2-overexpressing breast cancer cell line resistant to trastuzumab

We first tested the trastuzumab sensitivity of a human HER2 breast cancer cell line, BT474, in an in vitro cell viability test. The IC_50_ was 1.1 ± 0.2 μg/mL (Additional file 1: Figure S2). To obtain a trastuzumab-resistant cell line, we then exposed the cells to increasing sub-toxic concentrations of trastuzumab over a period of six months. The cell line remained viable at a concentration of 10 μg/mL. Trastuzumab resistance was achieved after 10 passages, since IC_50_ was still not reached. We called this resistant cell line “BT474-R”.

### Anti-HER2 gold nanoshells bind to BT474-R cancer cells in vitro

We checked that the BT474-R cell line was overexpressing HER2 at gene, mRNA and protein level (Fig. 2c). For the *HER2* gene, using DNA copy number, we found that the BT474-R cell line had a clinically relevant HER2 amplification with 10 copies of the *HER2* gene, while the MDA231 control cell line only had 2 copies. For mRNA, *HER2* was significantly overexpressed in the BT474-R cell line compared to the MDA231 cell line (RQ = 24 vs. 1, *P* < 0.05). For protein, we observed a typical HER2 membrane staining in the BT474-R cell line but not in the MDA231 cell line. To confirm that trastuzumab efficiently binds to HER2 membrane receptors on HER2-overexpressing BT474-R cells but not on MDA231 cells, we used fluorescent-labeled trastuzumab and found the same membrane pattern distribution in 100% of HER2-overexpressing BT474-R cells (Fig. 2d and Additional file 1: Figure S3). In addition, using dark field light microscopy, we showed an efficient binding of the anti-HER2 gold nanoshells to HER2 membrane receptors of all BT474-R cells analyzed (Fig. 2d and Additional file 1: Figure S3).

### Anti-HER2 gold nanoshells do not alter cell viability

In vitro cell viability tests for the nanoparticles, both functionalized and non-functionalized, showed no cell death on BT474-R and MDA231 cell lines exposed for 24 h, at increasing doses up to 10^14^ nanoshells/100 μL (Additional file 1: Figure S4A). Tissue toxicity was studied at 48 and 72 h after intravenous administration of 3.5 × 10^13^ gold nanoshells in mice. No visceral damage was detected microscopically, notably in bone marrow, liver, spleen or kidney (Additional file 1: Figure S4B).

### Gold nanoshells mainly accumulate in the liver and spleen

**Fig. 3:**
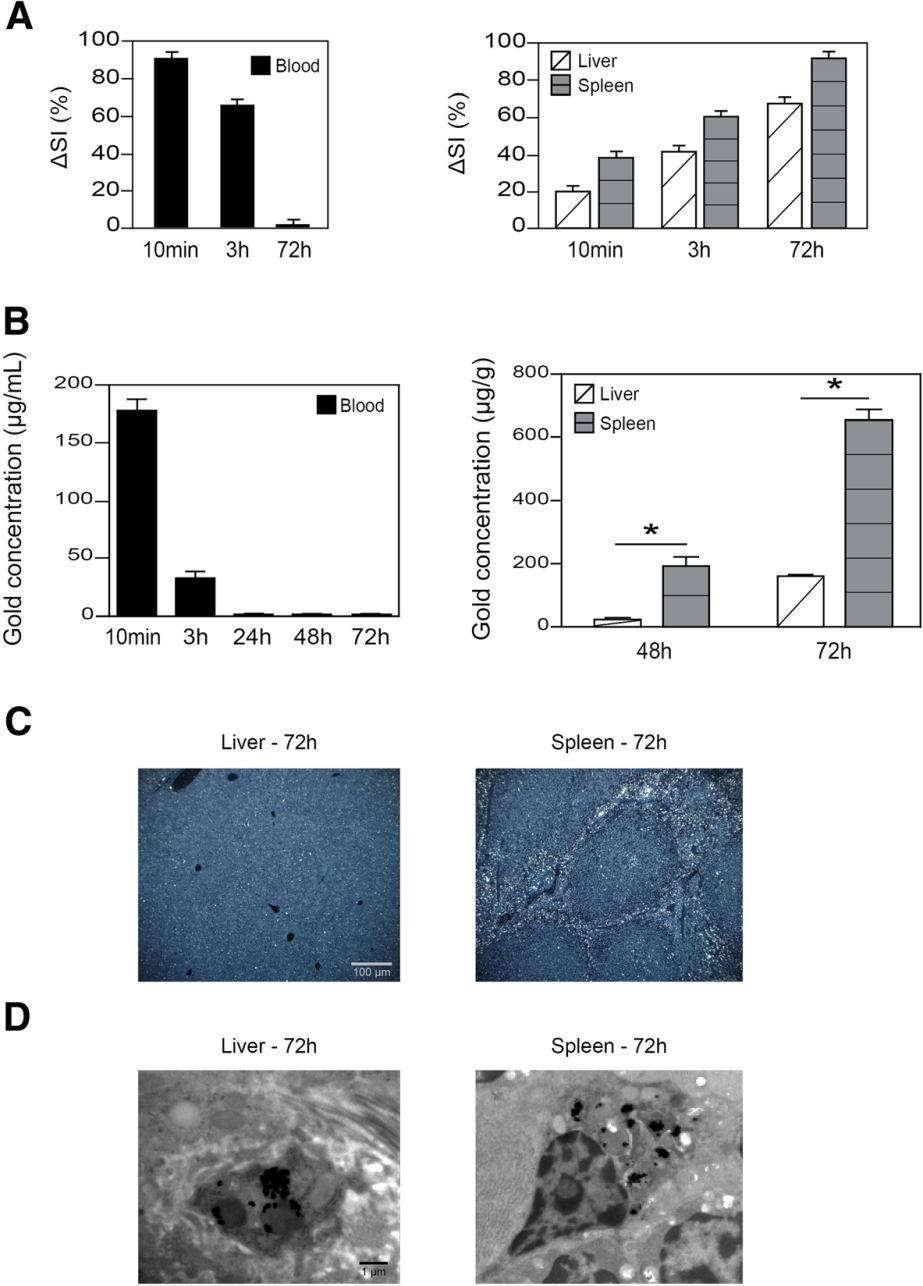
**a** Kinetic bio-distribution of anti-HER2 gold nanoshells (GNs) after intravenous administration of 3.5 × 10^13^ nanoshells in mice using magnetic resonance imaging (MRI). Results are expressed as percentages of initial signal variations (ΔSI), each signal intensity being compared with a pre-contrast signal. **b** Absolute gold concentration in blood and tissue samples after intravenous administration of 3.5 × 10^13^ anti-HER2 GNs using ICP-MS. Data is expressed as mean ± standard deviation, and comparisons between the liver and spleen concentrations were made using Student’s t-test (*P < 0.05). **c** Dark field images of tissue sections obtained from the liver and spleen after injection of anti-HER2 GNs. **d** TEM images of anti-HER2 GNs internalized in liver and spleen cells

To study the bio-distribution of the gold nanoshells in the mice, four complementary methods were used: inductively coupled plasma-mass spectrometry (ICP-MS), magnetic resonance imaging (MRI), dark field light microscopy and transmission electron microscopy (TEM). After intravenous administration of 3.5 × 10^13^ gold nanoshells, a blood peak of gold concentration was observed at 10 min with an absolute gold concentration of 181 ± 5 μg/mL using ICP-MS, and a 90 ± 2% difference with pre-contrast signal (ΔSI) using MRI. From 3 h onwards, we observed a rapid decrease in gold concentration, which became undetectable at 24 h. The MRI contrast signal was no longer detectable at 72 h. In the liver and spleen we observed a time-dependent accumulation using ICP-MS or MRI, mainly in the spleen (Fig. 3a and b). Using dark field light microscopy to analyze all organs, we confirmed that gold nanoshells mainly accumulated in the liver and in the spleen at 72 h. In addition, ultrastructural analyses enabled us to show that the nanoshells remained intact at 72 h, and predominantly accumulated in the macrophages of these two organs (Fig. 3c and d). No difference was observed in terms of organ bio-distribution between non-functionalized and functionalized anti-HER2 gold nanoshells.

### Anti-HER2 gold nanoshells efficiently target HER2-overexpressing cancer xenografts

**Fig. 4:**
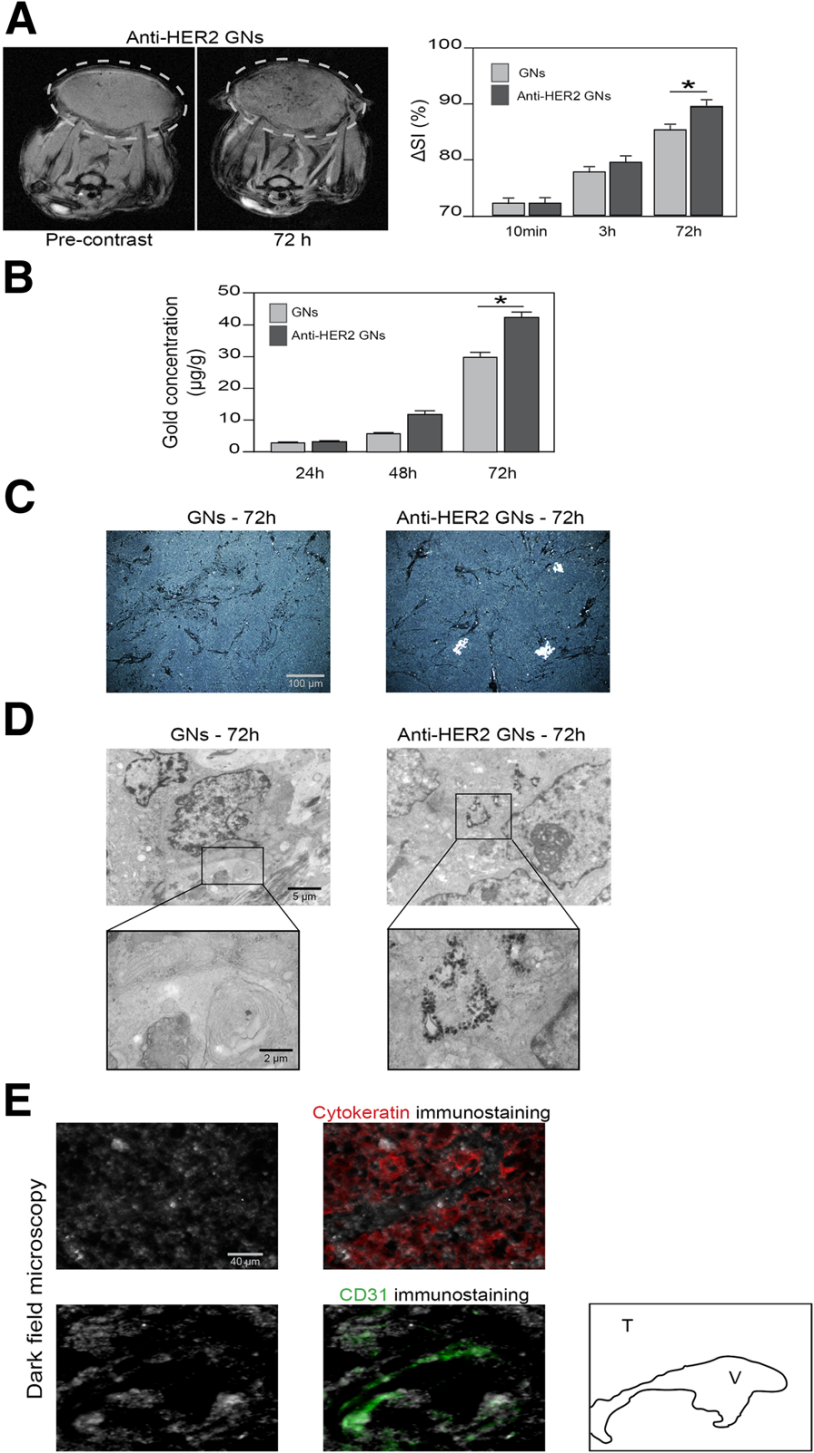
**a** T2-weighted magnetic resonance imaging of one xenografted tumor (circle) before and 72 h after intravenous administration of 3.5 × 10^13^ non-functionalized gold nanoshells (GNs) or anti-HER2 GNs, with corresponding histograms for percentage of signal initial variations (ΔSI). Each signal intensity is compared with a pre-contrast signal. **b** Absolute gold concentration in xenografted tumor over time after intravenous administration of non-functionalized gold nanoshells (GNs) or of 3.5 × 10^13^ anti-HER2 GNs using ICP-MS. Data is expressed as mean ± standard deviation, and comparisons between mice injected with non-functionalized GNs or with anti-HER2 GNs were made using Student’s t-test (*P < 0.05). **c** Dark field images of tumor sections at 72 h after injection of GNs. **d** TEM images of tumors at 72 h showing the presence of GNs within the cytoplasm of cancer cells. **e** Merged dark field images with immunofluorescence staining of human xenografted tumors 72 h after injection of anti-HER2 GNs, using anti-Cytokeratin (red) or anti-CD31 (green) antibodies (T: tumor; V: microvessel)

After analysis of xenografted tumors, we found a time-dependent accumulation of gold nanoshells up to 72 h. In addition, the bio-distribution in the tumor showed significantly higher gold concentrations with anti-HER2 gold nanoshells than with non-functionalized gold nanoshells, as evidenced by MRI: ΔSI was 89 ± 1% and 85 ± 1% respectively (*P* < 0.05, Fig. 4a). This result was confirmed by the ICP-MS analyses, showing significantly different absolute gold concentrations between the two types of nanoparticles with a 37% difference (41 ± 1 μg/g vs. 30 ± 1 μg/g, *P* < 0.05, Fig. 4b). Histological analyses also showed a preferential accumulation of anti-HER2 gold nanoshells in tumor xenografts at 72 h, in both tumor cells and endothelial cells (Fig. 4c). Ultrastructural studies demonstrated their cytoplasmic internalization (Fig. 4d). Using electron microscopy, for each tumor, we analysed 50 cells chosen because they were cut through the middle with an easily-recognisable nucleus. We then counted the mean number of gold nanoshells per cell at magnification 40,000. It was 109 ± 22 for mice injected with anti-HER2 GNs and 72 ± 13 for mice injected with non-functionalized GNs (*p* < 0.01). Using immunofluorescence staining coupled with dark field light microscopy, we showed that anti-HER2 gold nanoshells were co-localized with cancer cells. We also observed a co-localization of gold nanoshells with tumor endothelial cells (Fig. 4e). Overall, anti-HER2 functionalization improved the targeting of HER2-overexpressing human breast cancer cells in xenografts.

### Photothermal therapy induces cell death of BT474-R cells

When we assessed the effect of in vitro photothermal therapy after pulsed laser irradiation of BT474-R cells incubated with anti-HER2 gold nanoshells, we observed 95% cell death. Irradiation did not induce cell death in the absence of gold nanoshells (Additional file 1: Figure S5).

### Targeted photothermal therapy inhibits growth of trastuzumab-resistant xenografted tumors, through a collaborative anti-tumor and anti-angiogenic effect

**Fig. 5:**
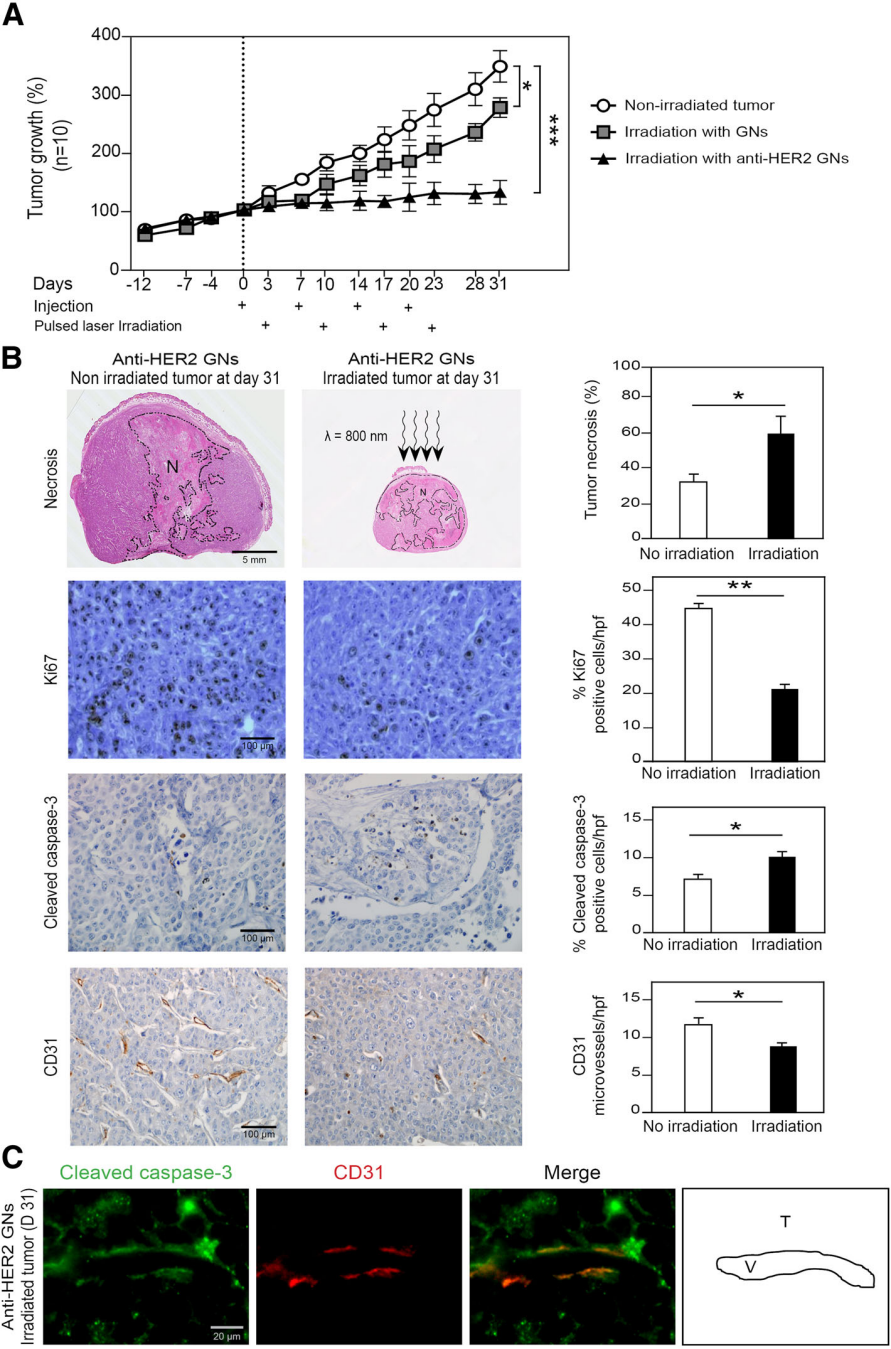
**a** Human xenografted tumor growth curves using mice grafted with trastuzumab-resistant HER2-overexpressing BT474-R cell lines. For each treatment group (non-irradiated tumors, irradiated tumors after intravenous administration of non-functionalized GNs, and irradiated tumors after intravenous administration of anti-HER2 GNs), 10 xenografted mice are used. Comparisons between the three treatment groups are made using ANOVA (* *P* < 0.05, *** *P* < 0.001). **b** Quantitative comparisons of tumor necrosis (N), cell proliferation, cell apoptosis and microvessel density between irradiated and non-irradiated tumors. Comparisons between groups are made using Student’s t-test (* *P* < 0.05, ** *P* < 0.01). **c** Double immunofluorescence staining of irradiated human xenografted tumor using anti-cleaved-caspase3 and anti-CD31 antibodies (T: tumor; V: microvessel)

For in vivo experiments, we grafted each mouse (*n* = 10) with two BT474-R tumors, one on each flank. We first confirmed their resistance to trastuzumab compared to mice xenografted with the BT474 cell line (Additional file 1: Figure S6). To mimic treatment schedules in patients, we injected 5 × 10^12^ anti-HER2 gold nanoshells in the mice once a week for four weeks. After each injection, one of the tumors was irradiated but not the other. On the basis of our preliminary pharmacokinetic study, we irradiated the tumors 72 h after the injection of the nanoshells, at the time of peak accumulation in the tumors. After four weeks of treatment, tumor growth inhibition was less marked for irradiated tumors using non-functionalized gold nanoshells compared to non-irradiated tumors. It was much more marked and complete for anti-HER2 GNs (*P* < 0.001, Fig. 5a). For each irradiated tumor, monitoring of temperature during photothermal treatment did not show any significant increase in macroscopic temperature over time. At histological level, the irradiation did not induce any cell damage on the skin surrounding an irradiated tumor (Fig. 5b).

To decipher the related biological effects, we assessed tumor necrosis, proliferation, apoptosis and microvessel density within the tumors at the time of euthanasia (Fig. 5b). We found an anti-angiogenic effect with large necrotic areas in irradiated tumors compared to non-irradiated tumors (58.2 ± 5% vs. 31.1 ± 2% respectively, *P* < 0.05), and a significant decrease in microvessel density in viable areas (8 vs. 12 microvessels/hpf respectively, *P* < 0.05). In addition, apparently viable tumor endothelial cells were apoptotic, co-expressing CD31 and cleaved caspase-3 (Fig. 5c).

We also showed that photothermal therapy induced a direct effect on cancer cells with significant inhibition of proliferation (21 ± 1% vs. 44 ± 1% Ki67-positive cells/hpf, respectively, *P* < 0.01), and significant tumor cell apoptosis (10 ± 1% vs. 7 ± 1% cleaved caspase3-positive cells/hpf, respectively, *P* < 0.05).

## Discussion

In this preclinical study, we overcame trastuzumab resistance in HER2-overexpressing breast cancer using anti-HER2 gold nanoshells and photothermal therapy. We purposely engineered a multimodal nanoparticle with three biological properties: an iron core for MRI, a gold shell for photothermal therapy, and an anti-HER2 antibody for active targeting of cancer cells. In a previous study on gold nanoparticles injected into xenografted mice, we had shown their preferential distribution in the liver and spleen [19]. Adding iron oxide and antibodies did not change the distribution profile in these organs, but immune-mediated targeting significantly increased the accumulation of functionalized gold nanoshells within the xenografted tumor, as previously reported [20–24].

One of the strengths of our study was that it designed the therapeutic protocol on the basis of a detailed pharmacokinetic study combining four complementary methods: ICP-MS, MRI, dark field light microscopy and TEM. We irradiated the mice 72 h after injection of anti-HER2 gold nanoshells, at the time of maximum accumulation in the tumors, to reach the maximum antitumor effect.

Most studies have reported empirical treatment protocols. After a search on PubMed using the algorithm “mouse AND gold nanoparticles AND cancer AND photothermal therapy”, we identified 256 articles among which 170 were within the scope of our study (Additional file 1: Figure S7 and Table S1). Eight of these 170 studies determined the time of laser irradiation on the basis of the results of a pharmacokinetic study. However, none of them used active targeting, resulting in smaller gold quantities within the tumors and lesser efficacy of the photothermal therapy [25].

Another original aspect of our study was that it used a femtosecond-pulsed laser. Only four of the 170 studies identified used pulsed lasers for photothermal therapy [26–29]. Femtosecond laser illumination is more cytotoxic in vitro than continuous-wave laser because short-pulse absorption produces higher sub-cellular temperatures [30]. In addition, the lower heat dissipation obtained with short pulses limits toxicity to healthy tissues surrounding the tumors [31], a required condition for a translational application to patients. As expected, we did not observe any increase in macroscopic temperature in the irradiated tumors and surrounding normal skin. The added value of femtosecond laser irradiation could be the combination of photothermal and photodynamic effects through the surface plasmon resonance of gold nanoshells, producing reactive oxygen species leading to cell apoptosis [32]. In our study, we demonstrated that femtosecond laser irradiation induced tumor endothelial cell apoptosis, with an anti-angiogenic effect and large necrotic areas within the irradiated tumors. This is coherent with the distribution of gold nanoshells within the lumen of vessels, and also in endothelial cells, which could be explained by the enhanced permeability and retention effect, a physical property enabling nanoparticles to accumulate passively within a tumor [8, 14]. Surprisingly, the anti-tumor effect remained limited using non-functionalized gold nanoshells, whereas it was total with anti-HER2 gold nanoshells. This suggests a major contribution of active targeting. The active targeting linked to functionalization explains the direct effect of irradiation on cancer cells, secondary to the internalization of the anti-HER2 gold nanoshells in their cytoplasm, as previously reported in vitro [33], and in HER2-overexpressing cancer xenografts sensitive to trastuzumab [34]. The large necrotic areas we observed may thus be the result of tumor vessel destruction, but also of cancer cell necroptosis secondary to the intense sub-cellular heat generated by photothermal therapy on targeted cells [35, 36].

## Conclusion

This preclinical study supports the use of anti-HER2 gold nanoshells and photothermal therapy to overcome trastuzumab resistance in HER2-overexpressing breast cancer. In addition, it opens the way to further research combining cytotoxic drugs with photothermal therapy.

## Additional file

Additional file 1: **Figure S1.** (**A**) T2 weighted MRI image of anti-HER2 GNs samples at five different iron concentrations (0.02 to 1 mM) at 7 Teslas and 25 °C. (**B**) Relaxation rates of GNs (red) and anti-HER2 GNs (green) according to the iron concentrations. At 7 Teslas and 25 °C, linear fitting of the data gives a relaxivity of 48.5 mM-1 s-1 for the GNs and 44.0 mM-1 s-1 for the anti-HER2 GNs. R2 represents the relaxation rate calculated as 1/T2, T2 being the transversal relaxation time. R2 is the coefficient of determination of linear regressions. (**C**) UV-Vis absorption spectra of anti-HER2 GNs (stock and administered suspensions) showing their photothermal stability under pulsed laser irradiation. **Figure S2.** Relative viability of BT474 and BT474-R cells incubated with different concentrations of trastuzumab. **Figure S3.** Immunostaining of BT474-R and MDA231 cells incubated with trastuzumab-FITC (green, left panel). Dark field images of BT474-R and MDA231 cells incubated with anti-HER2 gold nanoshells (right panel). **Figure S4.** (**A**) Relative viability of BT474-R and MDA231 cells incubated with different doses of gold nanoshells (GNs). (**B**) Hematoxylin and eosin staining of tissue sections obtained from bone marrow, liver and kidney after administration of anti-HER2 GNs: histological features in injected and saline-treated control mice were similar, with no abnormal phenotypic features. **Figure S5.** Relative viability of BT474-R and MDA231 cells incubated with anti-HER2 GNs or vehicle solution after pulsed laser irradiation (** *P* < 0.01). **Figure S6.** Tumor growth curves of the different groups of tumor-bearing mice during trastuzumab treatment (** *P* < 0.01). **Figure S7.** Flowchart of study selection. **Table S1.** list of the 170 studies for the literature search within the scope of our study. (PDF 3515 kb)

## Data Availability

All data generated or analysed during this study are included in this published article [and its supplementary information files].

## Abbreviations

GN: Gold nanoshell
HER: Human epidermal growth factor receptor-2
HPF: High power field
ICP-MS: Inductively coupled plasma-mass spectrometry
MPTMS: Mercaptopropyltrimethoxysilane
MRI: Magnetic resonance imaging
MTT: (3-[4, 5-dimethylthiazol-2-yl]-2,5-diphenyl-tetrazolium-bromide; Sigma)
OA: Oleic acid
OAm: Oleylamine
PEG: Polyethylene glycol
PVIS: Poly (1-vinylimidazole-co-vinyltrimethoxysilane)
PVP: Polyvinylpyrrolidone K12
RQ: Relation quantification
SD: Standard deviation
SDS: Sodium dodecylsulfonate
SE: Standard error
TEM: Transmission electron microscopy
TEOS: 1,2-dodecanediol, tetraethoxysilane
THPC: Tetrakis (hydroxymethyl) phosphonium chloride
