# Supplementary material for: Pulsed-laser irradiation of multifunctional gold nanoshells to overcome trastuzumab resistance in HER2-overexpressing breast cancer
Source: J Exp Clin Cancer Res. 2019 Jul 12;38:306. doi: 10.1186/s13046-019-1305-x (PMC6626398; doi:10.1186/s13046-019-1305-x)
Supplement: Supplementary file 1 — Figure S1. (A) T2 weighted MRI image of anti-HER2 GNs samples at five different iron concentrations (0.02 to 1 mM) at 7 Teslas and 25 °C. (B) Relaxation rates of GNs (red) and anti-HER2 GNs (green) according to the iron concentrations. At 7 Teslas and 25 °C, linear fitting of the data gives a relaxivity of 48.5 mM-1 s-1 for the GNs and 44.0 mM-1 s-1 for the anti-HER2 GNs. R2 represents the relaxation rate calculated as 1/T2, T2 being the transversal relaxation time. R2 is the coefficient of determination of linear regressions. (C) UV-Vis absorption spectra of anti-HER2 GNs (stock and administered suspensions) showing their photothermal stability under pulsed laser irradiation. Figure S2. Relative viability of BT474 and BT474-R cells incubated with different concentrations of trastuzumab. Figure S3. Immunostaining of BT474-R and MDA231 cells incubated with trastuzumab-FITC (green, left panel). Dark field images of BT474-R and MDA231 cells incubated with anti-HER2 gold nanoshells (right panel). Figure S4. (A) Relative viability of BT474-R and MDA231 cells incubated with different doses of gold nanoshells (GNs). (B) Hematoxylin and eosin staining of tissue sections obtained from bone marrow, liver and kidney after administration of anti-HER2 GNs: histological features in injected and saline-treated control mice were similar, with no abnormal phenotypic features. Figure S5. Relative viability of BT474-R and MDA231 cells incubated with anti-HER2 GNs or vehicle solution after pulsed laser irradiation (** P < 0.01). Figure S6. Tumor growth curves of the different groups of tumor-bearing mice during trastuzumab treatment (** P < 0.01). Figure S7. Flowchart of study selection. Table S1. list of the 170 studies for the literature search within the scope of our study. (PDF 3515 kb) [file 13046_2019_1305_MOESM1_ESM.pdf]

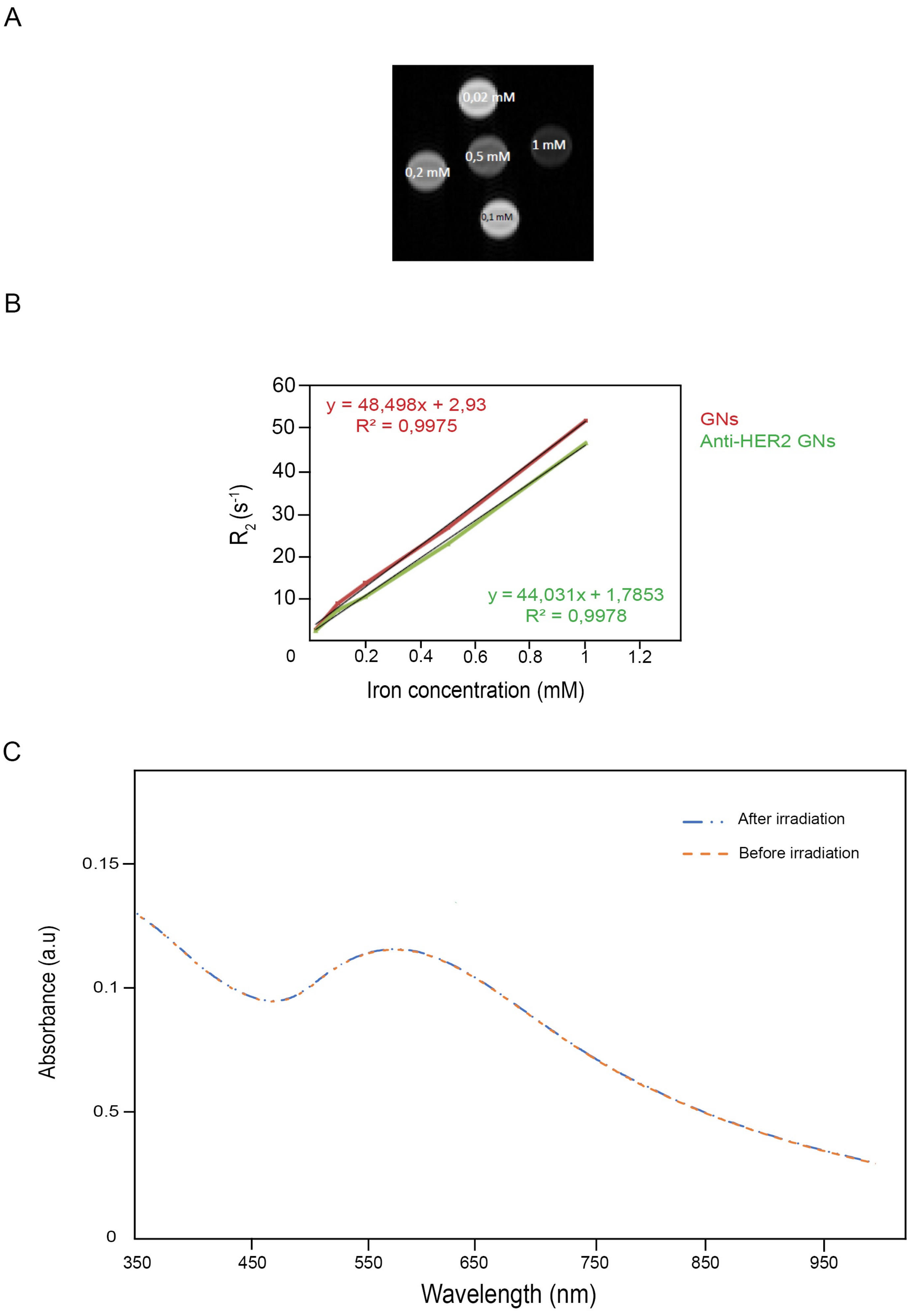
A
0,02 mM
1 mM
0,5 mM
0,2 mM
0,1 mM
B
y = 48,498x + 2,93
R² = 0,9975
GNs
Anti-HER2 GNs
y = 44,031x + 1,7853
R² = 0,9978
R2 (s-1)
0
10
20
30
40
50
60
0.2
0.4
0.6
0.8
1
1.2
Iron concentration (mM)
C
After irradiation
Before irradiation
Absorbance (a.u)
0.15
0.1
0.5
0
350
450
550
650
750
850
950
Wavelength (nm)

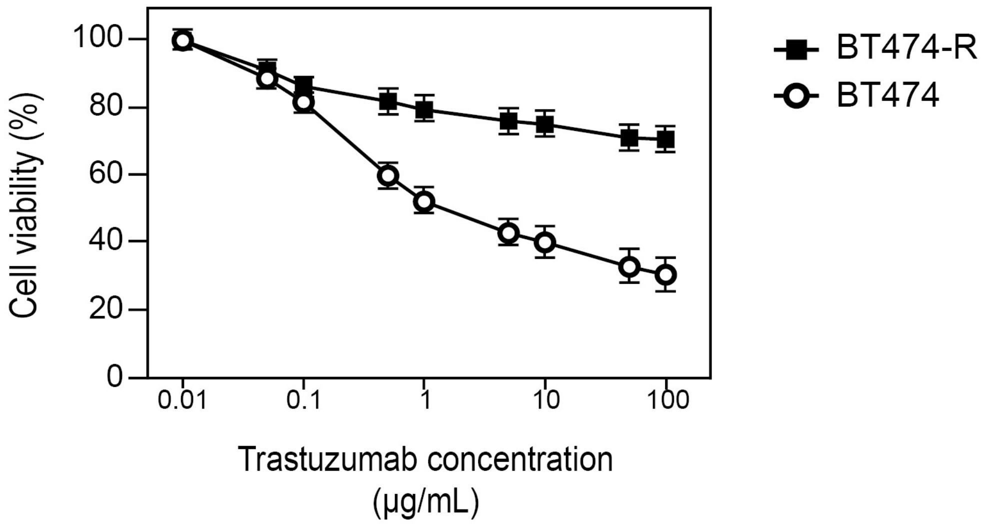
Cell viability (%)
100
80
60
40
20
0
0.01
0.1
1
10
100
Trastuzumab concentration
(μg/mL)
BT474-R
BT474

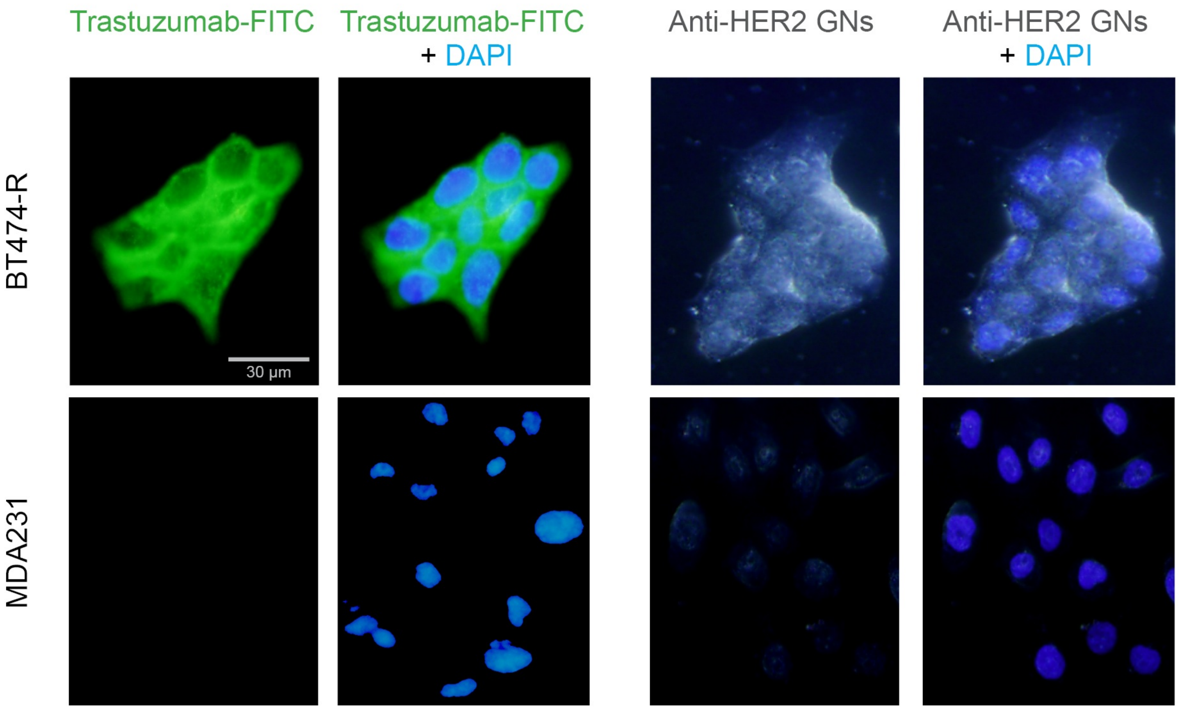
Trastuzumab-FITC
Trastuzumab-FITC
+ DAPI
Anti-HER2 GNs
Anti-HER2 GNs
+ DAPI
BT474-R
MDA231
30 μm

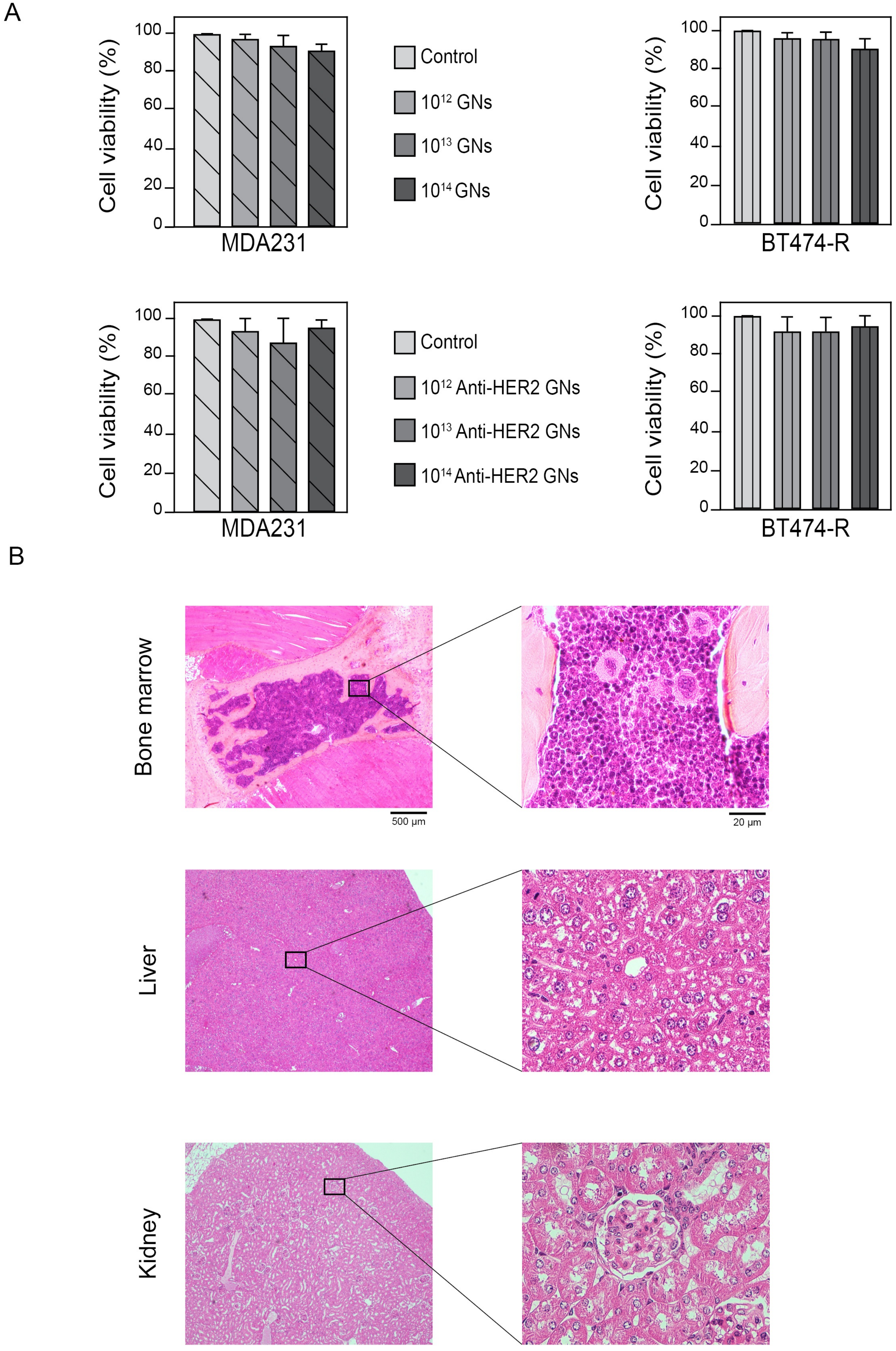
A
Cell viability (%)
100
80
60
40
20
0
MDA231
Control
10^{12} GNs
10^{13} GNs
10^{14} GNs
BT474-R
Control
10^{12} Anti-HER2 GNs
10^{13} Anti-HER2 GNs
10^{14} Anti-HER2 GNs
B
Bone marrow
Liver
Kidney
500 µm
20 µm

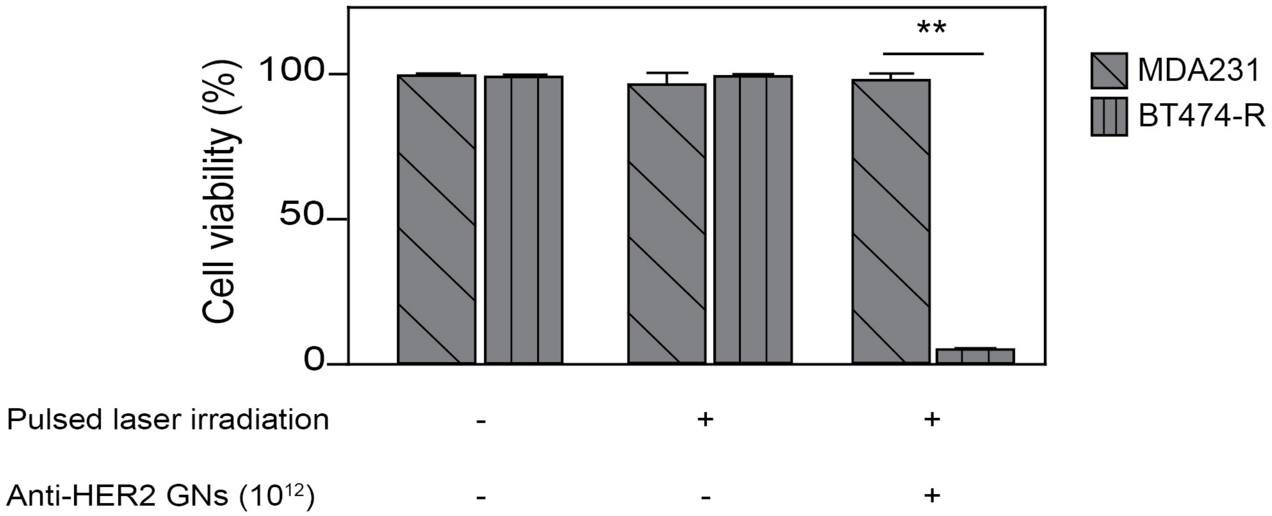
Cell viability (%)
100
50
0
**
MDA231
BT474-R
Pulsed laser irradiation - + +
Anti-HER2 GNs (10^{12}) - - +

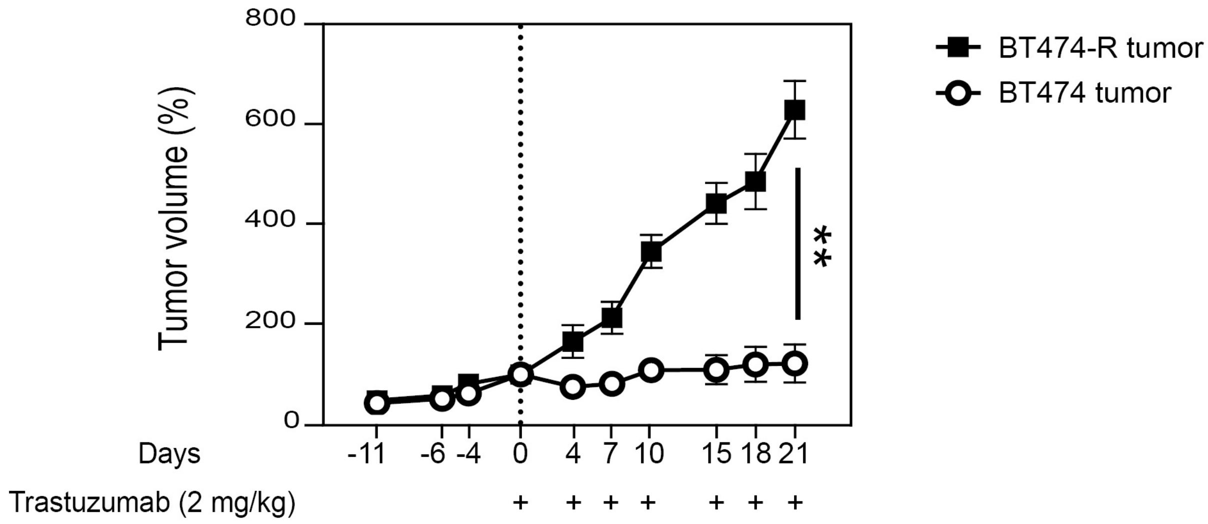

BT474-R tumor
BT474 tumor
Tumor volume (%)
800
600
400
200
0
**
Days
-11
-6
-4
0
4
7
10
15
18
21
Trastuzumab (2 mg/kg)
+ + + + + + +

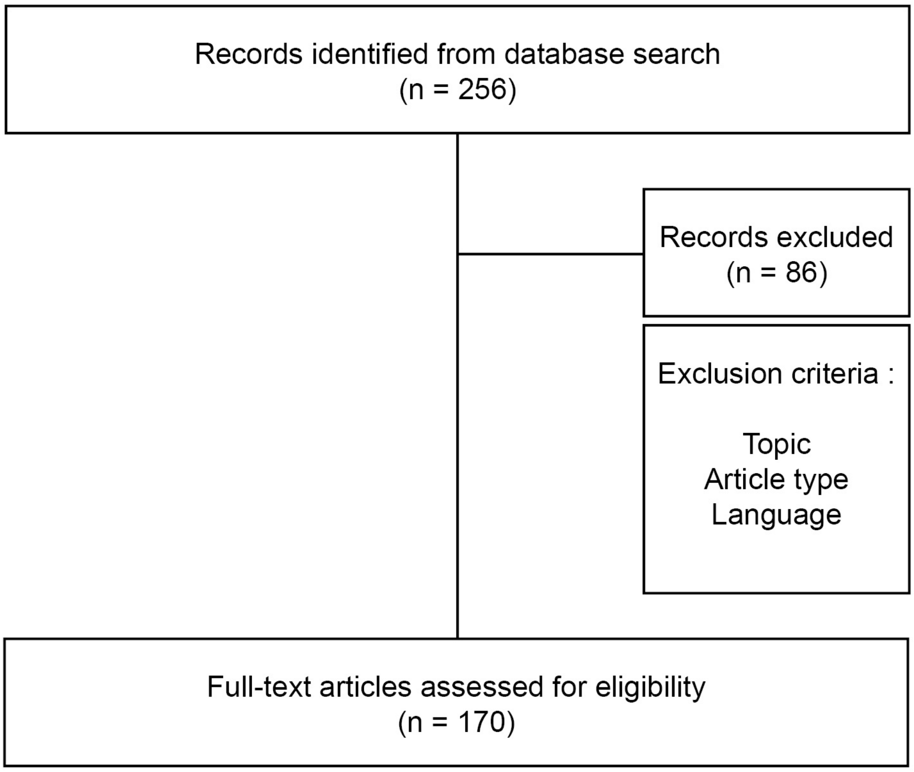
Records identified from database search
(n = 256)
Records excluded
(n = 86)
Exclusion criteria :
Topic
Article type
Language
Full-text articles assessed for eligibility
(n = 170)

Supplementary Figure Legends

Supplementary Fig. 1: (**A**) T2 weighted MRI image of anti-HER2 GNs samples at five different iron concentrations (0.02 to 1 mM) at 7 Teslas and 25 °C. (**B**) Relaxation rates of GNs (red) and anti-HER2 GNs (green) according to the iron concentrations. At 7 Teslas and 25 °C, linear fitting of the data gives a relaxivity of 48.5 mM-1 s-1 for the GNs and 44.0 mM-1 s-1 for the anti-HER2 GNs. R_2 represents the relaxation rate calculated as 1/T2, T2 being the transversal relaxation time. R^2 is the coefficient of determination of linear regressions. (**C**) UV-Vis absorption spectra of anti-HER2 GNs (stock and administered suspensions) showing their photothermal stability under pulsed laser irradiation.

Supplementary Fig. 2: Relative viability of BT474 and BT474-R cells incubated with different concentrations of trastuzumab.

Supplementary Fig. 3: Immunostaining of BT474-R and MDA231 cells incubated with trastuzumab-FITC (green, left panel). Dark field images of BT474-R and MDA231 cells incubated with anti-HER2 gold nanoshells (right panel).

Supplementary Fig. 4: (**A**) Relative viability of BT474-R and MDA231 cells incubated with different doses of gold nanoshells (GNs). (**B**) Hematoxylin and eosin staining of tissue sections obtained from bone marrow, liver and kidney after administration of anti-HER2 GNs: histological features in injected and saline-treated control mice were similar, with no abnormal phenotypic features.

Supplementary Fig. 5: Relative viability of BT474-R and MDA231 cells incubated with anti-HER2 GNs or vehicle solution after pulsed laser irradiation (** P<0.01).

Supplementary Fig 6: Tumor growth curves of the different groups of tumor-bearing mice during trastuzumab treatment (** P<0.01).

Supplementary Fig. 7: Flowchart of study selection.

Supplementary Table 1: list of the 170 studies for the literature search within the scope of our study

Autors	Journal	Year	Injection type	Repeated injections	PK study	Laser irradiation based on PK study	Delay between injection and irradiation	Laser type
Seo et al.	Colloid ans Surfaces B : Biointerfaces	2019	iv	no	yes	**yes**	4h or 8h	Continuous
Tsai et al.	ACS Applied Materials & Interfaces	2018	iv	no	yes	**yes**	30 min	Continuous
Zhou et al.	Acta Biomaterialia	2017	iv	yes	yes	**yes**	8h	Continuous
Song et al.	ACS Nano	2017	iv	no	yes	**yes**	24h	Continuous
Gao et al.	Biomaterials	2016	iv	no	yes	**yes**	6h	Continuous
Feng et al.	Nanoscale	2015	iv	no	yes	**yes**	2h	Continuous
Piao et al.	ACS Nano	2014	iv	no	yes	**yes**	48h	Continuous
You et al.	Particle and Fibre Toxicology	2014	iv	yes	yes	**yes**	24h	Continuous
Xu et al.	Adv Sci	2018	it	no	no	no	72h	**Pulsed**
Qin et al.	Nanoscale	2015	iv	no	yes	no	6h	**Pulsed**
Shao et al.	Scientific Reports	2013	iv	no	yes	no	8h	**Pulsed**
Lu et al.	Cancer Research	2010	iv	no	yes	no	6h	**Pulsed**
Liu et al.	Acta Pharm Sin B	2019	iv	no	no	no	24h	Continuous
Singh et al.	Nanotheranostics	2019	iv	no	no	no	24h	Continuous
Wang et al.	Nanoscale	2019	iv	No	no	no	24h	Continuous
Wang et al.	Int J Nanomedicine	2019	iv	Yes	no	no	Immediately	Continuous
Qin et al.	Int J Nanomedicine	2019	iv	no	no	no	Immediately	Continuous
He et al.	Nanoscale	2019	iv	no	no	no	6h	Continuous
Abed et al.	J Cancer Res Clin Oncol	2019	iv	no	no	no	24h	Continuous
Zhang et al.	Biomater Sci et al.	2019	iv	no	no	no	24h	Continuous
Mulens-Arias et al.	Nanoscale	2019	iv	no	no	no	24h	Continuous
Yang et al.	ACS Applied Materials & Interfaces	2019	iv	no	no	no	24h	Continuous
Zhang et al.	ACS Applied Materials & Interfaces	2019	iv	no	no	no	24h	Continuous

Elbialy et al.	Int J Pharm	2019	iv	no	no	no	immediately	Continuous
Liu et al.	Nanomedicine	2019	iv	yes	no	no	4h	Continuous
Chuang et al.	Int J Nanomedicine	2018	iv	no	no	no	24h	Continuous
Wang et al.	Int J Nanomedicine	2018	Peri-tumoral	no	no	no	48h and 8 days	Continuous
Lee et al.	Int J Pharm	2018	iv	no	no	no	4h	Continuous
Tu et al.	Colloids and Surfaces B : Biointerfaces	2018	iv	no	no	no	24h	Continuous
Zhang et al.	Chem Sci	2018	iv	no	no	no	24h	Continuous
Yan et al.	J Colloid Interface Sci	2018	iv	no	no	no	6h	Continuous
Li et al.	Theranostics	2018	iv	no	no	no	12h	Continuous
Wang et al.	Nanoscale	2018	iv	no	no	no	24h	Continuous
Cui et al.	J Am Chem Soc	2018	iv	no	no	no	immediately	Continuous
Xu et al.	Biomaterials	2018	iv	no	yes	no	24h	Continuous
Chang et al.	Nanoletters	2018	iv	no	yes	no	24h	Continuous
Yeo et al.	Nanoscale	2018	iv	no	yes	no	6h	Continuous
Kolovskaya et al.	Molecular Therapy Nucleic Acids	2017	iv	no	no	no	30 min	Continuous
Xu et al.	ACS Applied Materials & Interfaces	2017	iv	no	yes	no	24h	Continuous
Sun et al.	Theranostics	2017	iv	no	yes	no	4h	Continuous
Min et al.	Theranostics	2017	iv	no	oui	no	24h	Continuous
Zhao et al.	ACS Nano	2017	iv	yes	no	no	24h	Continuous
Yang et al	Bladder Cancer	2017	intravesical	yes	no	no	2h	Continuous
Chu et al.	Advanced Materials	2017	iv	no	no	no	24h	Continuous
Hu et al.	Journal of Controlled Release	2017	it	no	no	no	immediately	Continuous
Norregaard et al.	PLoS One	2017	iv	no	no	no	24h	Continuous
Gamal-Eldeenet al.	Biomedicine & Pharmacotherapy	2017	iv	no	no	no	/	Continuous
Zhang et al.	Journal of Photochemistry and Photobiology B: Biology	2017	iv	yes	no	no	48h	Continous

Wu et al.	Acta Biomaterialia	2017	it	yes	no	no	2h	Continous
Liu et al.	Theranostics	2017	iv	no	yes	no	24h	Continuous
Sun et al.	Biomaterials	2017	iv	no	yes	no	12h	Continuous
Nan et al.	ACS Applied Materials & Interfaces	2017	iv	yes	yes	no	24h	Continuous
Raghavan et al.	Nanomedicine	2017	it	no	yes	no	immediately	Continuous
Liu et al.	Nanomedicine	2017	it	yes	no	no	24h	Continuous
Chen et al.	Biomaterials	2017	iv	no	yes	no	12h	Continuous
Liu et al.	British Journal of Urology International	2017	it	no	no	no	24	Continuous
Parida et al.	Biochimica et Biophysica Acta	2017	iv	yes	no	no	/	Continuous
Chauhan et al.	European Journal of Pharmaceutical Sciences	2017	iv	yes	yes	no	30 min	Continuous
Zhang et al.	Theranostics	2017	it	no	no	no	24h	Continuous
Wei et al.	Advanced Healthcare Materials	2016	it	no	no	no	immediately	Continuous
Ge et al.	Biomaterials	2016	it	no	no	no	immediately	Continuous
Abo-Elfadl et al.	Journal of Photochemistry and Photobiology B: Biology	2016	it	no	no	no	immediately	Continuous
Gamal-Eldeen et al.	Journal of Photochemistry and Photobiology B: Biology	2016	iv	no	no	no	immediately	Continuous
Qian et al.	Analytical Chemistry	2016	iv	no	yes	no	24h	Continuous
Jørgensen et al.	Scientific Reports	2016	it	no	no	no	immediately	Continuous
Luo et al.	Small	2016	it	yes	no	no	immediately	Continuous
Li et al.	ACS Nano	2016	iv	no	yes	no	1h	Continuous
Chuang et al.	Biomaterials	2016	iv	yes	yes	no	1h	Continuous
Hu et al.	Small	2016	it	yes	no	no	10 min	Continuous
Xuan et al.	ACS Applied Materials & Interfaces	2016	iv	no	yes	no	20 min	Continuous
Deng et al	Nanoscale	2016	iv	yes	no	no	immediately	Continuous
Shi et al.	Nanoscale	2016	iv	no	yes	no	24h	Continuous
Wang et al.	Scientific Reports	2016	iv	yes	no	no	24h and 48h	Continuous
Sun et al.	Nanoscale	2016	it	no	no	no	1,5h	Continuous

Liu et al.	ACS Nano	2016	iv	no	yes	no	72h	Continuous
Piao et al.	ACS Applied Materials & Interfaces	2016	iv	no	no	no	24h	Continuous
Sun et al.	Advanced Materials	2016	iv	no	yes	no	1h	Continuous
Heidari et al.	Lasers in Medical Science	2016	it	no	no	no	immediately	Continuous
Wang et al.	Nanomedicine	2016	iv	no	no	no	24h	Continuous
Li et al.	Biomaterials	2016	it	no	no	no	48h	Continuous
Hao et al.	Journal of Controlled Release	2015	iv	yes	yes	no	4h	Continuous
Robinson et al.	Journal of Controlled Release	2015	iv	no	yes	no	24h	Continuous
McGrath et al.	ACS Nano	2015	it	no	no	no	30 min	Continuous
Yang et al.	Nanoscale	2015	it	no	no	no	1h	Continuous
Kang et al.	ACS Nano	2015	iv	no	no	no	72h	Continuous
Liang et al.	Small	2015	it	no	yes	no	2h	Continuous
Wu et al.	Oncotarget	2015	ip	no	no	no	immediately	Continuous
Liu et al.	ACS Applied Materials & Interfaces	2015	iv	no	yes	no	24h	Continuous
Zhang et al.	Biomaterials	2015	it	no	no	no	immediately	Continuous
Yu et al.	ACS Applied Materials & Interfaces	2015	iv	no	no	no	12h	Continuous
Zhou et al.	Journal of Biomedical Nanotechnolo gy	2015	it	no	no	no	immediately	Continuous
Kim et al.	Biomaterials	2015	it	no	no	no	24h	Continuous
Deng et al.	Advanced Materials	2015	it	no	yes	no	immediately	Continuous
Chen et al.	ACS Applied Materials & Interfaces	2015	iv	no	yes	no	24h	Continuous
Liang et al.	Theranostics	2015	iv	no	yes	no	immediately	Continuous
Liu et al.	Theranostics	2015	iv	no	yes	no	48h	Continuous
Tian et al.	Advanced Healthcare Materials	2015	it	no	no	no	immediately	Continuous
Liu et al.	Nanoscale	2015	it	no	no	no	24h	Continuous
Jin et al.	Colloids and Surfaces B : Biointerfaces	2015	iv	no	yes	no	1h	Continuous
Zhou et al.	Nanoscale	2015	iv and it	no	yes	no	24h	Continuous
Rengan et al.	Nanoletters	2015	iv	yes	no	no	48h	Continuous
Li et al.	Biomaterials	2015	it	yes	no	no	10 min	Continuous
Mooney et al.	Conference proceedings : Annual	2015	it	no	no	no	72h	Continuous

	International Conference of the IEEE Engineering in Medicine and Biology Society							
Shen et al.	Journal of Drug Targeting	2015	iv	yes	no	no	immediately	continuous
Green et al.	International Journal of Nanomedicine	2014	it	yes	no	no	immediately	continuous
Bai et al.	Journal of Biomedical Nanotechnology	2014	it	no	yes	no	immediately	continuous
Ayala-Orozco et al.	Journal of Controlled Release	2014	iv	no	yes	no	4h	continuous
Kwon et al.	Advanced Materials	2014	iv	no	yes	no	9h	continuous
Huang et al.	Molecular Pharmaceutics	2014	it	no	yes	no	24h	continuous
Li et al.	Advanced Healthcare Materials	2014	iv	yes	yes	no	24h	continuous
Wang et al.	Biomaterials	2014	it	yes	no	no	2h	continuous
Monem et al.	International Journal of Pharmaceutics	2014	iv	no	no	no	24h	continuous
Shi et al.	Nanoscale	2014	iv	no	yes	no	24h	continuous
Li et al.	Biomaterials	2014	iv	no	no	no	48h	continuous
Wang et al.	Scientific Reports	2014	iv	no	no	no	5 min (after sonoporation)	continuous
He et al.	Nature Communications	2014	it	no	no	no	immediately	continuous
Jing et al.	Biomaterials	2014	iv	no	yes	no	24h	continuous
Vankayala et al.	Biomaterials	2014	iv	no	no	no	immediately	continuous
Deng et al.	Theranostics	2014	it	no	no	no	immediately	continuous
Ayala-Orozco et al.	ACS Nano	2014	iv	no	yes	no	4h	continuous
Huang et al.	Journal of the American Chemical Society	2014	it	no	yes	no	immediately	continuous
Jing et al.	Theranostics	2014	iv	no	yes	no	24h	continuous
Kim et al.	IEEE Transactions on Ultrasonics, Ferroelectrics, and Frequency Control	2014	iv	no	yes	no	63 h	continuous
Choi et al.	Journal of Biomedical Optics	2014	iv	no	no	no	4h	continuous
Vankayala et al.	Small	2014	iv	no	no	no	4h	continuous

Nie et al.	Small	2014	iv	no	yes	no	6h	continuous
Liu et al.	ACS Applied Materials & Interfaces	2014	iv	no	yes	no	24h	continuous
Lin et al.	ACS Applied Materials & Interfaces	2014	it	no	no	no	immediately	continuous
Peng et al	Theranostics	2014	iv	no	no	no	4h	continuous
Ke et al.	Small	2014	it	no	no	no	immediately	continuous
You et al.	Pharmaceutical Research	2014	iv	yes	no	no	24h	continuous
Lee et al.	Biomaterials	2014	iv	no	yes	no	24h	continuous
Han et al.	International Journal of Nanomedicine	2014	it	no	no	no	immediately	continuous
Wang et al.	European Journal of Radiology	2014	it	no	no	no	immediately	continuous
Ke et al.	Theranostics	2013	iv	yes	yes	no	1h	continuous
Lee et al.	Journal of Controlled Release	2013	it and iv	no	yes	no	immediately	continuous
Chen et al.	Biomaterials.	2013	iv	no	yes	no	8h	continuous
Bagley et al.	ACS Nano	2013	iv	no	no	no	48h	continuous
Shen et al.	Nanoscale	2013	iv	no	yes	no	24h	continuous
Chen et al.	Theranostics	2013	iv or it	yes	yes	no	8h	continuous
Liu et al.	ACS Nano	2013	iv	no	yes	no	24h	continuous
Bear et al.	PLoS One	2013	it	no	no	no	5 min	continuous
Yang et al.	Biomaterials	2013	iv	no	yes	no	36h	continuous
Shi et al.	Biomaterials	2013	it	no	no	no	immediately	continuous
Jin et al.	Biomaterials	2013	it	no	no	no	immediately	continuous
Kirui et al.	Nanomedicine	2013	iv	yes	yes	no	12	continuous
Lin et al.	ACS Nano	2013	it	no	no	no	immediately	continuous
Wang et al.	Advanced Materials	2013	it	no	yes	no	4h	continuous
Nam et al.	ACS Nano	2013	iv	yes	yes	no	24h	continuous
Wang et al.	ACS Nano	2013	iv	no	yes	no	72h	continuous
Gormley et al.	Journal of Controlled Release	2013	iv	no	yes	no	48h	continuous
Elbialy et al.	Journal of Biomedical Nanotechnology	2013	it or iv	no	yes	no	5 min (it) - 24h (iv)	continuous
Yuan et al.	Nanomedicine	2012	iv	no	no	no	48h	continuous
Khlebtsov et al.	Langmuir	2012	iv	no	yes	no	24h	continuous
Choi et al.	Small	2012	iv	no	yes	no	6h	continuous
You et al.	Journal of Controlled	2012	iv	yes	yes	no	24h	continuous

	Release							
Melancon et al.	Journal of Controlled Release	2011	iv	yes	yes	no	24h	continuous
Lu et al.	Cancer Research	2011	iv	no	yes	no	24h	continuous
Day et al.	Journal of Neuro-Oncology	2011	iv	no	yes	no	24h	continuous
Choi et al.	ACS Nano	2011	iv	no	yes	no	24h	continuous
Melancon et al.	Investigative Radiology	2011	it	no	yes	no	24h	continuous
Elsherbini et al.	International Journal of Nanomedicine	2011	it	no	no	no	30-60 min	continuous
Xie et al.	International Journal of Nanomedicine	2011	iv	no	no	no	24h	continuous
Chen et al.	Small	2010	iv	no	yes	no	72h	continuous
Gobin et al.	Small	2010	iv	no	yes	no	24h or 48h	continuous
Li et al.	Molecular Pharmaceutics	2010	iv	no	yes	no	6h	continuous
Park et al.	Proceedings of the National Academy of Sciences of the United States of America	2010	iv	no	no	no	72h	continuous
Goodrich et al.	Journal of Biomedical Optics	2010	iv	no	yes	no	24	continuous
Maltzahn et al.	Cancer Research	2009	iv	no	no	no	72h	continuous
Lu et al.	Clinical Cancer Research	2009	iv	no	yes	no	4h	continuous
Lal et al.	Accounts of Chemical Research	2008	iv	no	yes	no	18h	continuous
